# Supplementary material for: Are carbon emissions trading and green financial instruments synergistic? -Comprehensive quantitative research based on content analysis
Source: PLoS One. 2024 Mar 7;19(3):e0298601. doi: 10.1371/journal.pone.0298601 (PMC10919601; doi:10.1371/journal.pone.0298601)
Supplement: S1 Data — (ZIP) [file pone.0298601.s001.zip › Data/地级市Z_F工作报告.zip!/║┌┴·╜¡/╦τ╗»╩╨/└┤╫╘╣1⁄2╓┌║┼í╛┬φ┐╦╩2╛▌═°í┐_2019.docx]

各位代表:

现在，我代表市人民政府，向大会报告工作，请予审议，并请各位市政协委员提出意见。

一、2018年工作回顾

2018年，是外部环境发生深刻变化，经济下行压力最大的一年；是推进供给侧结构性改革，向高质量发展迈出坚实步伐的一年；也是“三大攻坚战”取得重大成效的一年；更是打基础、补欠账，解决历史遗留问题最多的一年。这一年，我们在习近平新时代中国特色社会主义思想指引下，在省委、省政府和市委的坚强领导下，紧紧依靠全市人民，牢牢把握“都城地”建设目标定位，埋头苦干，攻坚克难，完成了既定任务，朝着全面建成小康社会又迈进了一大步。

一年来，我们始终保持战略定力，顶住下行压力，紧紧抓住结构优化、动能转换、质量提高这个关键，全市经济稳中向好，好中有升。面对主要指标低迷开局，我们强力调度，以月保季、以季保年，一路追赶。预计地区生产总值增长6%，固定资产投资增长5.5%，社会消费品零售总额增长6.6%，公共财政预算收入增长8.8%，规上工业增加值增长8.5%，继续在全省综合类城市中保持靠前位置。民营经济占国民经济份额超过60%，产业项目投资占固定资产投资48.3%，食品和农副产品精深加工业占比达58.2%，企业技改投入增长71.7%，新增高新技术企业9家。收入质量显著提高，税收占财政收入比重68.2%。城乡居民收入增速高于经济增速。市经开区创新驱动、动能转换、产业升级实现新跨越，主营业务收入和公共财政预算收入分别增长22%和77%，运行质量明显提高，示范引领和辐射带动作用明显增强。黑龙江（海伦）大豆交易中心被明确为全省动能转换项目。庆安奥利达奈德获省政府质量提名奖。这些积极深刻变化，让我们对未来发展更加充满信心。

一年来，我们牢牢把握农业供给侧结构性改革这条主线，加快培育农业农村优势产业，农村经济发生可喜变化。粮食总产达到235亿斤，实现“十五连丰”。积极引导农民和其他经营主体不断提高市场敏感度，主动调整种植结构，一些市场销路好、我们有优势的农产品得到了较快发展。蔬菜等高效作物面积增长10.3%，鲜食玉米增长33%，产量15亿穗，在全国地级市面积最大、品质最佳、品牌最响、效益最好，连续两年成功举办中国鲜食玉米大会，被确定为全国鲜食玉米产业示范区，获批国家农业科技园区，庆安首批通过国家现代农业产业园认定。“兰明青望海”蔬菜产业扶贫经济带开工超5000万元项目20个，农民人均增收2665元，兰西被确定为全国（蔬菜）绿色高产高效创建示范县。庆安承办的首届中国农民丰收节黑龙江分会场庆祝活动，办出了绥化特色、龙江精神、中国风采，庆安大米连续被评为中国大米十大公用区域品牌。农民和企业质量意识、标准意识、品牌意识、市场意识明显增强。龙头企业和合作社带基地、闯市场，大宗作物订单达到30%，蔬菜订单达到65%，“五谷杂粮下江南”活动被确定为“2018年中国区域农业品牌十大营销案例”，“寒地黑土”品牌入选2018中国区域农业品牌影响力十强，农村一二三产实现融合发展。青冈被确定为全国农村三产融合发展先导区，承办全省“粮头食尾”“农头工尾”现场会。秸秆养牛、牛奶生产走在全国前列。特色物产之乡再添新成员，明水被授予“中国黑豆之乡”和“中国干菜之乡”。

一年来，我们围绕构建具有绥化特色的产业体系，充分挖掘放大绥化优势潜力，招大商、上项目、促投资，主导产业方向更加清晰。跳出绥化看绥化，在国内国际产业分工中找准发展定位，全力做好“三篇大文章”，紧扣“粮头食尾”“农头工尾”“油头化尾”，加快推进“1+4+N”现代产业体系，签约产业项目745个，总投资980.6亿元，浙江新和成、阿里巴巴、网易这些战略投资者和国内、世界500强企业越聚越多。京粮生物、万达广场等15个项目投产运营，省重点产业项目开复工率和投资完成率均居全省前三位。总投资70亿元的黑龙江新和成玉米生物发酵产业园在2平方公里的土地上全面铺开，一期土建工程基本完工，设备安装日夜不停，一个站在世界玉米产业链顶端的企业正在由蓝图变为现实，向全球玉米生物发酵中心目标快步迈进。与已经投产和正在建设的中粮、龙凤、京粮、成福、象屿、国投、昊天等，形成了具有比较优势和竞争优势的玉米产业链，成为推动绥化发展的强大引擎。生物质新能源产业成长迅猛，计划装机总容量达到178.5兆瓦，中能建、华能、九洲电气等热电联产项目接连落地，海伦地势坤生物质热电联产项目投产，让农作物秸秆释放出更大发展能量。中俄天然气管道主管线焊接全部完成。安达被确定为全省“油头化尾”战略实施主体功能区和全国化工产业论坛永久会址。有项目才有未来，只要我们始终把招商引资和产业项目建设作为经济发展生命线，绥化就大有奔头！

一年来，我们抓住主要矛盾和主攻方向，全力打好“三大攻坚战”，为决胜全面建成小康社会打下更加坚实的基础。坚决落实党中央重大决策部署，持续抓重点、补短板、强弱项。坚持县域经济、龙头企业、合作组织、村民集体共同推进，5个国贫县均已形成优势主导产业，都有立县支柱企业。开展贫困户“千元增收行动”，精准滴灌，点对点推行金融、光伏、电商、“三大五小”等25个增收项目，产业扶贫到户覆盖率98%，提高23个百分点。发放扶贫小额贷款总量全省最多，贫困户扶贫小额保险参险率100%。打赢了危房改造和饮水安全“两个歼灭战”。望奎、绥棱摘帽，全市贫困发生率降至0.94%，又有5.07万人从贫困中走出来。明水健康扶贫工作全国领先。全市脱贫攻坚入选“大国智慧、中国样板”脱贫攻坚典型案例。以前所未有的政策密度、工作力度、攻坚强度，大打蓝天、碧水、净土保卫战。实现市本级燃煤小锅炉“清零”，5个区域供热站淘汰燃煤锅炉接入集中供热。严格落实五级网格和“四包五定”机制，秸秆禁烧至今“零火点”，守住了在全省不烧第一把火的底线。大力推进秸秆综合利用，秸秆离田、翻埋、沤肥、压块、发电全面推进，全市秸秆综合利用率达到75%以上，农业农村部秸秆综合利用现场会在海伦召开。在全省率先开展用脚步丈量河流，河湖长制全面落地，一大批污水处理、畜禽粪污资源化利用、水源地保护项目开工建设。海伦黑土地保护模式得到国家充分肯定。全市空气质量优良天数达标率由86.7%提高到90.6%。市环保局获国家先进集体。我们用实际行动诠释了环境就是民生，青山就是美丽，蓝天也是幸福。政府债务总体可控、安全平稳。强化监管穿透、风险隔离，一批非法集资案件被消灭在初始阶段。金融支持地方经济发展能力增强，与省证监局合作建立的金融工作站直接融资成效明显。天有为主板上市进入辅导期。加大金融对外合作与创新力度，与富士康达成战略合作协议，未来将向我市提供超百亿元融资担保。富士康全资子公司富金服科技有限公司在我市设立的商业保理公司，是该公司在东北设立的第一家企业，也是黑龙江省第二家商业保理公司。

一年来，我们坚持用统筹理念和系统思维，推进城乡发展，谋划重大基础设施项目，努力让人民群众从每一天的变化中感受到幸福。拓展城市发展空间，增设宝华新区申请已报国务院。从增强功能、提升品位、改善人居入手，实施“城市双修”，持续提高城乡建设水平。广顺东街改造全面完成，花园街正式通车，一批城市道路建设相继完成，拉开了绥化城区快捷通畅交通格局建设序幕。新建和完善升级唯实公园、唯恋公园等11处绿地广场公园，改造升级金华街市场。新增停车位700多个。二次供水改造等惠及3万户。坚决向顽固的不法利益宣战，清理违建5.2万平方米，还城市以美丽。加大村镇建设投资力度，26个国家级重点镇和6个特色小镇完成投资46亿元。农村人居环境整治全面铺开。“厕所革命”试点到乡到村。青冈获批国家地质公园建设资格。安达入围全国百佳深呼吸小城。建设改造国省干线、农村公路1404公里。望奎被评为“四好农村路”全国示范县。绥巴路完成投资85%。阁山水库大坝合龙，呼兰河北部联通、民用机场、绥大高速等重大项目前期工作有序推进。北大荒通用航空肇东往返佳木斯135短途运输航线正式开航，圆了绥化多年的飞翔之梦。

一年来，我们以开放的思维、开阔的视野，向改革要动力，向开放要活力，向创新要竞争力，绥化发展站在了新的起点上。顺应时代潮流，努力在更高起点、更高层次、更高目标上推进改革、扩大开放。全年完成13个领域123项改革任务。土地确权登记证书发放率超过95%，农村土地“三权分置”改革成效显著，安达“三块地”改革试点、肇东昌五和庆安平安国家农业产业示范强镇、海伦土地增减挂结余指标省外流转、北林农村“双创”、庆安新型经营主体建设、绥棱农村集体产权制度改革走在全省全国前列。铁路“三供一业”移交有序推进。与阿里巴巴、网易严选签订战略合作协议，天猫绥化农产品官方旗舰店正式运营，我市被商务部正式确定为供应链试点城市。深度融入“一带一路”，主动参与“龙江丝路带”，打通对俄蔬菜出口通道。与湛江全面开展“北粮南下，南产北上”、蔬菜深加工及冷链物流、军粮供应、铁海联运合作。参加首届进博会成果斐然，肇东大庄园签单12.86亿美元，占全省37%。外贸进出口总额增长114%，绥化海关正式获批，亚麻纺织基地升格为国家外贸转型升级基地，肇东被确定为全省唯一的中国国际农产品加工产业园。我们相信，只要敢闯敢试，一个更加开放、更具活力、更有魅力的绥化一定会成为现实！

一年来，我们致力于强保障、惠民生、促和谐，积极回应群众关切，下决心解决痛点难点问题，发展成果更广泛惠及全市人民。城镇登记失业率控制在4%以内，就业形势持续向好。城乡居民基本养老保险覆盖面进一步扩大，低保和特困供养人员救助标准全面提高。市本级棚改开工2009套，超额完成省定任务。又有2.21万农户扒掉危房住进新房。义务教育均衡发展深入推进，教师违规补课行为得到有效遏制。基本医疗保险实现异地就医直接结算，卫生健康服务能力稳步提升，药品采购“两票制”网上监管填补全省空白。寒地黑土特色文化感染力进一步增强，扶贫小品《扶起来》登上央视。体育基础设施建设更加完善，全民健身基础保障水平明显提高。安全生产主要指标全部下降，全年没有发生重特大事故。打掉黑社会性质组织犯罪集团2个，恶势力集团团伙38个，扫黑除恶走在全省前列。

一年来，我们下大力气推进转思想、转职能、转作风，政府公信力、执行力大幅度提升。把政治建设贯穿政府工作全过程，树牢“四个意识”，坚定“四个自信”，坚决做到“两个维护”。在优化营商环境中呼唤思想解放，服务企业的新和成模式在省电视台新闻联播连续三期专题报道，成为我们招商引资的金字招牌。加大“放管服”改革力度，市本级90%以上的行政许可和公共服务事项实现“最多跑一次”。创新工作方式方法，向标准看齐，用制度规范，重点工作专班推进，制定招商引资规则，完善政府工作制度，行政工作更加规范。开展“法治建设年”活动，深化法治政府建设，政府领导出庭应诉成为常态。海伦、庆安获“全国法治县”创建活动先进单位。强力开展政府失信违诺专项清理，全国信用排名前移102位。加强对重要领域、重点岗位的监督约束，“三公”经费支出压缩10%以上。一年来，我们还以雷霆手段，打赢了房地产领域信访积案化解、巡视整改、环保督察、秸秆禁烧、大棚房和违法占地清理、燃煤锅炉改造、抗击特大洪水等多场硬仗，政府应对危机和突发事件的能力显著提升，在关键大事上立场鲜明，态度坚决，守住了底线。尤其是投入2亿多元，解决了多年得不到回迁安置、多次到省进京集体上访等历史遗留难题，市区889户全部得到了妥善安置，还上了历史欠账，党和政府的威信得到了极大提升。

一年来，我们还有力推进了民族宗教、市场监管、国防动员、妇女儿童、统计监测、审计监督、扶残助残、市志档案、气象人防、老龄双拥慈善各项工作，实现了新的发展。

各位代表！2018年是改革开放40周年。这一年，我们乘着改革开放的强劲东风，圆满完成了各项任务。回望改革开放这40年，我们创造了绥化发展史上最为广泛而深刻的伟大奇迹，书写了最为波澜壮阔的壮丽篇章。绥化40年取得的历史性成就和历史性变革，源自于党的正确领导，源自于人民对美好生活的不懈追求。在此，我代表市人民政府，向全市人民，向各级人大代表、政协委员，向各民主党派、工商联、无党派人士和人民团体，向中省直单位、驻绥部队、公安民警、武警官兵和社会各界，表示衷心的感谢！向改革开放40年来为绥化发展进步做出贡献的所有人士，致以崇高的敬意！

在看到成绩的同时，我们也清醒地看到存在的差距和不足。一是经济总量小，发展速度慢，发展质量不高，内生动力不足，发展要素集聚能力不够，发展潜力没有得到充分释放，实现高质量发展的拐点还没有形成。二是城乡之间、县域之间、产业之间发展不充分、不平衡的问题突出，还没有形成整体区域竞争优势，有的市县没有找到正确的发展思路和有效路径，全市没有一个全国百强县，有些县在全省的位次始终没有大的提升；具有竞争力的新兴产业集群还不够多、不够大，产业链短、价值链低的问题还比较突出。三是对经济发展规律和阶段性特征的把握还不够，对影响经济全局的重点工作抓的不狠、不到位、不持续，特别是对招商引资和产业项目建设重视不够，投放力量不足，效果不明显，没有形成蓬勃发展的态势。四是基础设施和民生欠账较多，环保压力较大，一些短板还没得到很好补齐，由群众合理诉求解决不到位引发的社会矛盾和隐患依然存在。五是一些政府工作人员思想守旧，闯劲不足，作风不实，担当不够，缺少加快发展、高质量发展的紧迫感和危机感，抓经济工作的手段、服务企业和群众的能力还有很大差距。

总体上，我市还处于依靠投资拉动和消费带动发展阶段，处于爬坡过坎、攻坚克难的艰难期，打基础、调结构、转动能的关键期，推动全面振兴全方位振兴的奋进期。我们要正视问题，坚定信心，增强定力，一步一个脚印，踏踏实实地向前迈进。

二、2019年工作安排

各位代表！2019年是新中国成立70周年，是决胜全面建成小康社会关键之年，我们站在了一个新的历史关口。做好今年的工作，对于未来几年绥化发展的走向，对于赢得高质量发展的主动权至关重要。我们深感责任重大，也深知前路艰辛，但发展的愿望和期盼，从未像今天这样浓烈；发展的底气和士气，从未像今天这样强劲；发展的信心和决心，从未像今天这样坚定。我们坚信，只要全市人民万众一心、众志成城，就一定能够抓住机遇、创造未来、铸就辉煌！

2019年政府工作的总体要求是：以习近平新时代中国特色社会主义思想为指导，深入贯彻落实党的十九大、中央经济工作会议和省委十二届四次全会、省委经济工作会议、市委四届五次全会精神，聚焦贯彻落实习近平总书记在深入推进东北振兴座谈会重要讲话和考察黑龙江时的重要指示精神，统筹推进“五位一体”总体布局，协调推进“四个全面”战略布局，坚持稳中求进工作总基调，坚持新发展理念，坚持推进高质量发展，坚持以供给侧结构性改革为主线，深入解放思想，深化市场化改革，扩大高水平开放，坚决打好“三大攻坚战”，统筹推进稳增长、促改革、调结构、惠民生、防风险工作，推动“都城地”向更高质量发展，为全面建成小康社会收官打下决定性基础，开创绥化全面振兴全方位振兴新局面。

按照市委全会部署，2019年主要预期目标是：地区生产总值增长6%；固定资产投资增长5%；规模以上工业增加值增长6%；社会消费品零售总额增长6%以上；公共财政预算收入增长4%左右；外贸进出口总额增长7%；实际利用外资增长3%；单位GDP能耗与上年持平；城镇和农村常住人口人均可支配收入均增长6%以上。

重点做好七项工作：

（一）始终把招商引资和产业项目建设作为促进经济发展的生命线，形成大抓项目、大招项目、大建项目的强烈态势。抓发展是政府第一要务，抓项目就是抓发展。要做实做强做优实体经济，瞄准“三个第一、三个千亿”的努力方向，全力做好“三篇大文章”，构建“1+4+N”现代产业体系，实施“千百十”工程，尽快形成多点支撑、多业并举、多元发展新格局。聚焦新一轮产业项目三年行动计划，培育壮大特色主导产业。一个好的产业可以兴起一座城市，一个好的项目就是一个新的经济增长点。要坚持以市场换投资、以产品换链条、以资源换项目。玉米产业要力促总投资70亿元的新和成项目一期工程、投资46亿元的象屿等项目投产，总投资20亿元的星湖科技落地，促成玉米加工实现千万吨产能、千亿级产出。汽车电子产业要启动建设中国北方汽车电子产业园，做好天有为电子主板上市推动，到2020年形成1000万台（套）生产能力，成为全国最大汽车仪表加工基地。蔬菜产业要围绕生产基地、冷链物流、批发市场，突出蔬菜、菌类营养功能成分提取开发，开工建设一批产业化项目，扩大“北菜南销”核心区影响力。实施大豆产业振兴计划，启动海伦大豆中国特色农产品优势区、大豆产业园区、大豆种子研究所、永和农场建设，黑龙江大豆交易中心投入运营，在大豆种子繁育、种植、加工、品牌、销售等关键环节上破题，打造全国最大的非转基因大豆生产加工基地和高端豆粉集散地。汉麻产业力争启动汉麻交易中心，打造集原料种植、生产加工、产品开发、检验检测、市场开拓于一体的汉麻产业发展平台。下大力气发展俄气产业，提高工业化利用比重。支持中再生发展汽车后产业。完成烟厂异地搬迁。调整利益分配方式，发展“飞地经济”，集中打造大型专业化产业园区。加强庆安医药产业园、安达石化产业园、国家农业科技园区、肇东国际农产品加工产业园建设。加大“百大项目”推进实施力度，滚动谋划生成储备总投资2亿元以上重点产业项目50个，开工建设固定资产投资2亿元以上重点产业项目40个，总投资1亿元以上重点产业项目建成投产40个，每个县（市、区）和经开区至少引进投资5亿元以上项目2个。打好招商引资硬仗。坚持把招商引资作为“一号工程”，加快构建“大招商”格局。依托资源优势和市场优势，聚焦重点产业、重点资源、重点区域开展务实招商。绘制主导产业图谱，提升产业链水平。注重招大引强，瞄准世界500强、国内100强、行业10强企业，尤其是紧盯国内食品行业龙头企业，定向精准招商。重大项目实行“一班人负责、一竿子到底、一口气干成”包保机制。争取有更多项目进入省“百大项目”，实际利用省外资金增长10%以上。开展开发区改革升级行动。实行市场化运行、企业化管理、绩效化考核、专业化招商，激活开发区活力，既“筑巢引凤”，又“固巢留凤”，全面提升产业集群和创新引领能力。加大各类开发区补短板力度，市经开区要启动铁路专用线、热电联产等项目建设。全市盘活撂荒项目和僵尸企业一半以上。新增规上企业20户，战略新兴企业达到40户以上。完善项目服务保障、督查考核问责机制。树立以项目论成败、以招商论英雄的工作导向，定期研判、定期调度、定期研究要素配置补齐短板。加强项目各环节不间断跟踪，建立重大项目市县分层管理制度。建立“专班+园区”“目标+考核”的招商引资新机制。以企业需求为目标，以好项目、大项目落地为标准，在项目生成和服务保障中形成更多产业，为绥化全面振兴全方位振兴积累更厚的物质基础。

（二）深入实施乡村振兴战略，加快农业强市建设步伐。没有农业农村现代化，就没有整个国家现代化。坚持农业农村优先发展，努力打造农业强、农村美、农民富的“绥化样板”。深入推进农业供给侧结构性改革。落实粮食安全市长责任制，稳定粮食综合产能，进一步优玉米、减水稻、增大豆、扩蔬菜，鲜食玉米要发展到80万亩。在标准化生产、规模化经营、品牌化创建、法制化监管上实现重点突破，建立一批高质量特色基地，绿色、有机食品认证面积达到1260万亩，“三减”面积950万亩。促进农产品精深加工高质量发展。围绕“粮头食尾”“农头工尾”提档升级谋划生成资源精深加工项目，向产业链中高端延伸，向研发设计和品牌营销两端延伸。建设全程质量控制、清洁生产和可追溯体系。支持精深加工企业将企业总部和加工产能向县城和中心镇转移。继续办好农民丰收节、鲜食玉米节、绿色有机水稻文化节，积极组织新型经营主体参加各类农产品展洽活动。规划创建以农业产业为主体、旅游为先导、文化为灵魂、乡村振兴为目标的田园综合体项目10个。启动阿里巴巴、网易未来农场建设。提高“兰明青望海”蔬菜产业扶贫经济带建设水平。以高标准特色蔬菜品种核心示范区为引领，蔬菜面积达到160万亩，开工产业链项目50个以上，投产一批加工、冷藏项目。5个县市分别建设千亩规模“十化”标准园区10个、万亩以上标准园区1个。加强畜牧产业能力体系建设。构建长效机制，坚决打好非洲猪瘟疫情处置歼灭战和防控攻坚战，切实研究和解决生猪产业面临的实际问题，确保生猪产业健康稳定发展。坚持走绿色化发展、产业化带动、规模化养殖、科技化引领、市场化营销、社会化服务的畜牧产业发展路子。加快奶业振兴步伐。肉蛋奶加工率达到30%以上。打好秸秆综合利用硬仗。突出新技术、新装备和新工艺的示范推广，以肥料化、饲料化、原料化为主导，综合利用率要达到90%以上。开工建设生物质热电联产项目4个，消化秸秆180万吨，翻埋还田200万亩以上，地头沤肥30万亩以上。庆安银泉造纸扩产要实现较大突破。补齐农业基础设施短板。制定农业基础设施建设中长期规划，建立农业项目库。扎实推进高标准农田建设。继续做好十六道岗水库前期工作。开工建设呼兰河及其主要支流治理工程和中小河流治理工程。完成阁山水库工程建设。推进农村饮水安全巩固提升工程，实现自来水村村通。开展强村富民行动。采取市场化办法，经营盘活农村集体机动地、水面、闲置房屋等资产资源，发展农村集体资产合作社、土地合作社、农村劳动力合作社，培育现代农业强村50个以上、农产品加工强村20个以上、特色旅游强村10个以上、电子商务强村10个以上，壮大农村集体经济。要深入挖掘农业内部增收潜力，支持引导农村劳动力外出就业、就地就近就业、返乡创业，不断增加财产性和转移性收入。开展好农村人居环境整治行动。持续深化学习浙江“千村示范、万村整治”经验。严格执行“十有十化六无”标准，每个县（市、区）确定2个示范乡镇，按照三类行政村整治标准各确定2-3个示范村。全市行政村生活垃圾治理率达到70%以上，畜禽养殖废弃物综合利用率达到72%以上，庭院经济示范村增长10%以上。加强村屯绿化美化净化，倡导乡风文明。完成三类行政村整治目标40%。让农村有乡愁可记忆，有幸福可守望。

（三）坚定实施创新驱动发展战略，挖掘和培育新的经济增长点。创新是引领发展的第一动力。坚持以创新为引领，全面提升创新能力，加快形成新动能。强力推进科技创新。实施新一轮“科技型企业三年行动计划”。启动建设绥化、海伦等6个省级以上农业科技园区。着力培育科技型中小企业、高新技术企业、创新型领军企业，新增高新技术企业5户以上，在孵企业达到170户。持续深入推进大众创业、万众创新。细化创新创业支持政策，每个县（市、区）都要创建一批创业型示范乡镇(街道)、示范村（社区）、示范平台。加快发展新业态新商业模式。深化与网易严选、京东、天猫合作，电子商务交易额达到80亿元，网络零售额达到14亿元。继续推进松花江都市田园创新示范区建设，启动最具中国北方冰雪特色高品位旅游小镇项目，绥棱田园养生示范县建设要取得较大突破。实施文旅企业倍增计划，扩大安达马术节、青冈猛犸象故乡影响力，提高品牌辨识度。全市旅游综合收入增长13%以上。推进质量兴市、品牌强市。把质量提升摆在更加突出位置，制定品牌培育创建实施方案，建立绥化名优特产品目录，强化地方特色标准，推进有机产品和地理标志产品示范区建设，设立市政府质量奖。深入实施“寒地黑土+”双品牌战略，扩大品牌影响力，升级“五谷杂粮下江南”活动，加大进平台、进展会、进高校、进社区、进商超力度，重点发展上海、北京、杭州旗舰店、体验店，在数量、规模、影响力上实现大的突破。积极参加龙江好粮油中国行、哈洽会、绿博会等活动，继续推广“寒地黑土”系列好产品，朝着把“寒地黑土”打造成超千亿的农产品区域公用品牌目标迈进。开展绥化“十大名小吃”评选活动。创新需要更多更好的人才来支撑。重点做好“引才”“育才”“用才”三篇文章，实施好“头雁”行动和“万里挑一”计划，编制急需紧缺人才引进指导目录，健全完善重点人才库，建立人才公寓。用产业聚人才，用平台育人才，用事业留人才，让更多千里马在绥化大地上竞相奔腾、大显身手。

（四）坚决打好“三大攻坚战”，夯实全面建成小康社会基础。打好“三大攻坚战”是重大政治责任，也是决胜全面建成小康社会的底线任务和必须迈过的坎。我们要集中力量，打好重点战役，确保完成全年攻坚任务。坚决打好精准脱贫攻坚战。脱贫攻坚是一场大考。要把脱贫攻坚作为重大政治任务和第一民生工程来抓，严格执行现行扶贫标准，全面提高脱贫质量。在兰西、明水摘帽的基础上，实现海伦、青冈摘帽，44个贫困村出列，2.96万贫困人口脱贫，贫困发生率降至0.2%。主攻产业扶贫，实施贫困户“人均千元增收行动”，产业扶贫到户全覆盖。重点解决好“两不愁三保障”面临的突出问题，持续补齐短板弱项，关注非贫困县、非贫困村和非贫困人口中的“边缘户”，防止返贫和新的贫困发生。坚决打好污染防治攻坚战。深入抓好中央环境保护督察问题整改。按照市统一规划、县组织实施的原则，3月底前编制完成全市城乡垃圾和污水处理布局规划。坚决打赢蓝天保卫战。从优化产业结构、能源结构、运输结构、用地结构入手，深化秸秆禁烧、面源污染治理、燃煤小锅炉淘汰三大行动，严禁市本级建成区内新建35蒸吨以下燃煤锅炉，确保完成省下达的空气质量考核指标。坚决打赢碧水保卫战。用脚步继续丈量河流，落靠河湖长制“有名”“有实”，向河湖“四乱”和水污染宣战，还河湖一个整洁干净空间。加强湖库和饮用水水源地保护，持续改善全市水环境质量。年底前完成北郊和经开区两个污水处理厂建设，两年内完成县城污水处理设施提标改造、国家级重点镇和呼兰河流域涉及乡镇污水处理设施建设。坚决打赢净土保卫战。摸清土壤污染底数，加强耕地土壤环境分类管理，实施土壤管控与修复，加大黑土地保护力度，开展涉重金属企业专项整治行动，强化固体废物污染防治。肇东生活垃圾发电厂要在年内投产，启动望奎、青冈生活垃圾焚烧发电项目，两年内“户分类、村收集、乡转运、县处理”的体系要基本建成。落实黑土地保护项目90万亩，完成绿化造林10万亩。坚决打好防范化解重大风险攻坚战。加强政府债务源头管控，化解存量，控制增量，严防变量，守住不发生系统性区域性风险的底线。严厉整治非法集资和金融乱象，严密防控互联网金融风险。畅通银行、证券、保险、期货服务经济发展渠道，探索设立产业引导基金、专项投资基金，扩大融资规模。基本完成农信社改制。引导企业进一步强化诚信意识和规则意识，营造良好金融生态环境。

（五）高质量推进以人为核心的新型城镇化，有效提高城镇承载能力。城镇建设水平，不仅关系居民生活质量，而且也是城市生命力所在。要突出产业、特色、生态三大重点，走出一条产业兴、人才聚、人口旺、活力足的新型城镇化发展之路。发挥好规划的重要引领作用，谋划城市功能定位和空间布局。以打造哈尔滨都市圈核心区副中心城市为目标，按照“多规合一”的空间规划要求，重点启动融入哈尔滨一小时两小时经济圈规划、哈绥铁客专外溢效应对产业功能拉动规划、宝华区建设规划。提升城市建设水平。加快建设中心城市，做好“城乡双修”，城区道路、广场、公共绿地建设要体现经济价值与民生价值的统一，加快“环城林、通水系、镶嵌绿地广场”规划建设，打造田园生态城市。绥巴路通车，绥胜南路、文明路等道路贯通。持续开展老旧小区改造，特别是供热管网改造。推进“三供两治”建设，启动建设绥化市红兴水库呼兰河城区水源工程、三水厂及供水管线改造、城区地下水资源保护、城区污泥处理、二次供水改造等重点工程，让市民吃上更加安全的饮用水。抓好城市棚户区改造，解决好城市低收入和困难家庭的住房问题。在全市范围内统筹建设生活垃圾焚烧发电厂，实现垃圾就近处理。积极推进重点镇建设和特色小镇培育，每个县（市、区）要打造2-3个产业特色鲜明、要素集聚、宜居宜业、富有活力的特色小镇。创新城市治理方式。廓清市区事权财权关系，属地与属事相称，城市综合执法与社区、物业有机结合，提高服务水平。重拳开展“城市去疤行动”，城区存量违法建筑“清零”。开展市容环境整治行动，对违规占道经营、城市“三乱”、道路交通、门脸牌匾、广告进行集中整治。启动3D数字城市建设。加强重大基础设施建设。抓住国家加大基础设施等领域补短板力度的窗口期，扩大有效投资。全力配合做好哈绥铁客专、哈绥北黑铁路提速改造、绥化经北安至加格达奇铁路规划建设，以及哈肇高速肇东段项目开工工作。继续做好绥化民用机场前期工作。积极融入哈尔滨都市圈环线路网建设。抓好绥大高速公路建设推进工作。加快推进县乡村道路改造升级，提高“四好农村路”比重。使绥化逐步成为名副其实的全省交通枢纽。

（六）持续深化改革开放，有效激发内生动力和发展活力。坚持把深化改革开放作为绥化全面振兴全方位振兴的根本动力。实施好政府机构改革。3月底前全面完成各项任务，释放更多“红利”。推进重点领域改革。深化供给侧结构性改革，在“巩固、增强、提升、畅通”上下功夫。更多运用市场化、法治化手段，深化现代农业综合配套改革，推进农村土地改革、农村集体产权制度改革、集体经营性资产股份制改革、农事服务体制机制改革，重点解决农技、农机、畜牧等涉农服务职能弱化、工作断层、力量薄弱问题。落实好第二轮土地承包到期后再延长30年政策。推进城投、农投混合所有制改革，启动国有资本投资、运营公司试点。推进经济发达镇改革试点。打造营商环境的“绿水青山”。重塑环境、重振雄风。大踏步推进以“最多跑一次”为重点的“放管服”改革，以“放”为原则，以“不放”为特例。全面实行市场准入负面清单制度。固化推广新和成模式，推行“马上办、一次办、我帮办”。推进“证照分离”“多证合一”和“四零”承诺服务创建。坚持“双随机、一公开”常态化。对破坏发展环境的人和事，一定依法依规从严从重处理。谁砸坏绥化营商环境的牌子，绥化就一定砸掉谁的饭碗！要实质性降低企业负担，减少审批环节。上半年全市企业开办时间压缩到8个工作日以内，不动产登记限5日内办结。努力把绥化打造成为行政许可审批全省流程最优、时间最短、设限最少、效能最高的城市。支持民营经济发展。坚持“两个毫不动摇”，激发和保护企业家精神。继续推进中小企业发展三年行动计划，建立“专精特新”中小企业库，多措并举为民营企业提供金融支持。加强对口交流合作。全面落实绥化湛江合作框架协议，在产业园区、产品销售、招商引资上提升合作层次。特别要立足湛江，把目光投向更广阔的粤港澳地区。打造多元化开放平台。主动融入“一带一路”，积极参与“龙江丝路带”，扩大以对俄为重点的经济合作。加强同日、韩、蒙等东北亚地区的经贸往来。加快出口基地建设，拓宽对外贸易渠道。创建国家级出口农产品质量安全示范区。启动绥化海关建设，加快绥化（庆安）保税物流中心建设，推进对外贸易便利化。

（七）更加关注补齐民生领域短板，让人民群众共享振兴成果。民生是最大的政治。着力践行以人民为中心的发展思想，在幼有所育、学有所教、劳有所得、病有所医、老有所养、住有所居、弱有所扶上取得新进展。要更加充实百姓获得感。优化教育资源配置，推进义务教育优质均衡发展，谋划设立绥化职业教育学院。深化以高考改革为重点的教育改革。实施第三期学前教育三年行动计划。坚决完成义务教育发展基本均衡县国检验收任务。强力整顿师德师风，办公平优质教育。实施更加积极的就业政策，城镇登记失业率控制在4.3%以内。完善社会保障体系。实施全民参保计划，积极推进医疗保险、养老保险提标扩面，推行“互联网+社保”。关爱退役军人，加强农村留守儿童关爱保护和城乡困境儿童保障,让困难群众心中有温暖、生活有奔头。要持续提升百姓幸福感。推动文化繁荣昌盛，加强文明城市创建，提高城乡文化活动场所建设水平，创作更多带露珠、沾泥土的文化精品，讲好绥化故事。加快建设健康绥化。继续深化公立医院改革，推进分级诊疗制度建设，创建一批省级健康乡镇、健康村、健康社区，推行“互联网+医疗健康”。广泛开展全民健身活动，谋划建设市文体中心，全市新建各类全民健身工程100处，其中市本级10处以上。要不断增强百姓安全感。深入开展“法治建设年”活动，建设平安绥化。坚持发展新时代“枫桥经验”，健全完善公共法律服务体系建设。强力推进扫黑除恶专项斗争，全面实施“雪亮工程”，推进智慧城市建设。下更大力气抓好食品药品安全、特种设备安全。坚决打好信访积案化解攻坚战，给历史遗留问题划上圆满句号。持之以恒抓好安全生产，加快构建风险分级管控和隐患排查治理双重预防机制。我们要始终把人民群众的小事当成大事，让全市人民深深体会到生活在绥化是幸福的，也是有尊严的！

三、全面加强政府自身建设

打铁必须自身硬。顺应伟大新时代，满足人民新期待，完成全年目标，必须顺势而为、奋发向上，革故鼎新、守正出新，打开新局面，展现新作为。

第一，毫不动摇地把政治建设摆在首位。心中有信仰，行动有力量。牢固树立“四个意识”，坚定“四个自信”，坚决做到“两个维护”，做政治上的明白人。以理想信念立根铸魂。进一步提高政治站位，严守政治纪律政治规矩，坚决贯彻市委决策部署。

第二，坚定不移地继续解放思想。思想是行动的先导。产业要转型，思想先转型；产业要升级，思维先升级；动能要转换，思路先转换。深入开展解放思想推动高质量发展大讨论，从灵魂深处闹革命，革除一切妨碍发展的思维方式、做法规定、体制机制，消除不问责不落实、小问责小落实、大问责大落实的思维惯性和工作惯性，搬走权威、自我、习惯三块大石头，根除“七大顽症”，交出改革开放再出发绥化答卷。

第三，驰而不息锤炼担当过硬作风。重任在肩，不容懈怠。我们每一名干部都没有任何资格在人民群众的困难面前闭上眼睛，转过身去。要紧盯群众关切，把群众的“表情包”作为检验作风的“晴雨表”。要持续强化能力培训和实践锻炼,提高抓落实、解决实际问题和应急处理能力，增强适应新时代要求的新本领，适应岗位要求。要强化“新官都要理旧账”的要求，以强烈的使命感，不驰于空想，不鹜于虚声，一级压一级、一级抓一级、一级逼一级，把定下的事情干出精彩。要不看痕迹看实绩，坚决整治表态多调门高、行动少落实差的形式主义、官僚主义，大兴调研之风，紧盯短板弱项，一锤接着一锤敲，让美好蓝图一步步变为现实。

第四，持之以恒依法行政为民履职。认真坚持“三重一大”事项报告制度。自觉接受人大的依法监督、政协的民主监督和社会的舆论监督，坚决执行人大及其常委会的决议决定，认真办理人大代表建议、政协委员提案。推行行政执法公示制度、执法全过程记录制度、重大执法决定法制审核制度、法律顾问制度、智库咨询制度、信访案件合法性律师审查制度、被诉行政机关负责人出庭应诉制度、失信惩戒制度，提高决策水平和执行力。抓紧补上民生和环境欠账。我们决不干后人给我们买单，给后人添乱的事情。政府热线统一纳入“12345”市长公开电话运行管理，完成“互联网+政务”平台建设，助推最多跑一次向一次不用跑、办事不求人转变。

第五，坚持不懈推进党风廉政建设。落靠“一岗双责”，公共资源项目全部入场交易，“三公”经费只减不增，办公用房和公务用车管理更加规范。严格执行各项规定，建立亲清政商关系，对腐败现象露头就打。抓小节就是立大德。政府系统干部特别是领导干部要带好头、打好样，干干净净为人民做事。

各位代表！新时代是奋斗者的时代。山再高、往上攀，总能登顶；路再长、向前走，必定到达！让我们更加紧密地团结在以习近平同志为核心的党中央周围，在市委的坚强领导下，改革开放再出发，推动“都城地”建设向更高质量发展，奋力走好绥化全面振兴全方位振兴新路子，以优异成绩庆祝新中国成立70周年！
